# Divergent Chemical Cues Elicit Seed Collecting by Ants in an Obligate Multi-Species Mutualism in Lowland Amazonia

**DOI:** 10.1371/journal.pone.0015822

**Published:** 2010-12-30

**Authors:** Elsa Youngsteadt, Patricia Guerra Bustios, Coby Schal

**Affiliations:** 1 Department of Entomology and W.M. Keck Center for Behavioral Biology, North Carolina State University, Raleigh, North Carolina, United States of America; 2 Facultad de Ciencias Biológicas, Universidad Nacional de San Antonio Abad del Cusco, Cusco, Perú; University of California Davis, United States of America

## Abstract

In lowland Amazonian rainforests, specific ants collect seeds of several plant species and cultivate them in arboreal carton nests, forming species-specific symbioses called ant-gardens (AGs). In this obligate mutualism, ants depend on the plants for nest stability and the plants depend on ant nests for substrate and nutrients. AG ants and plants are abundant, dominant members of lowland Amazonian ecosystems, but the cues ants use to recognize the seeds are poorly understood. To address the chemical basis of the ant-seed interaction, we surveyed seed chemistry in nine AG species and eight non-AG congeners. We detected seven phenolic and terpenoid volatiles common to seeds of all or most of the AG species, but a blend of the shared compounds was not attractive to the AG ant *Camponotus femoratus*. We also analyzed seeds of three AG species (*Anthurium gracile*, *Codonanthe uleana*, and *Peperomia macrostachya*) using behavior-guided fractionation. At least one chromatographic fraction of each seed extract elicited retrieval behavior in *C. femoratus*, but the active fractions of the three plant species differed in polarity and chemical composition, indicating that shared compounds alone did not explain seed-carrying behavior. We suggest that the various AG seed species must elicit seed-carrying with different chemical cues.

## Introduction

In the ant-garden (AG) mutualism, arboreal ants collect seeds of specific epiphytic plants and cultivate them in nutrient-rich carton nests. As the seeds germinate and grow, the nests become hanging gardens. AG interactions are obligate for both ant and plant participants, and occur in the tropical Americas and Southeast Asia [1], [2], [3]. The basis for ant recognition of AG seeds, however, is poorly understood in any AG interaction.

AGs have been studied repeatedly in lowland Amazonia, where the ant-plant mutualism comprises a diverse but specific fauna and flora of at least five ant species in four subfamilies, and at least 15 epiphyte species in seven families [1], [3], [4]. The interaction between AG ants and seeds differs from other ant-seed interactions in its greater species-specificity and the subsequent long-term symbiosis of the participating species [1], [5]. AG ants are also outstanding for their abundance and behavioral dominance. In lowland Amazonia, foraging territories of AG ants can occupy more than one third of forest area, depending on habitat type, and AG ants are the most frequently encountered and numerically abundant species in arboreal ant samples [1], [6], [7]. The success of AG ants, like other dominant arboreal ant species, has been attributed to their independence from pre-existing nesting substrates or nesting space, which allows them to exploit the most resource-rich microhabitats [1], [7]. In the case of AG ants, this independence is inseparably linked to the epiphytic mutualism, because the long-term structural integrity of the large carton nests depends on AG plants, which dry the nest by transpiration and provide shelter from heavy rains [8], [9].

Similarly, AG species are among the most abundant epiphytes in lowland Amazonia, and are almost never found thriving outside of ant nests [1], [10]. In lowland rainforests where epiphytes are limited by substrate and nutrients, AGs are the most important habitat for vascular epiphytes, due to the porous texture and enriched N, K, and P of AG carton [11], [12], [13]. Association with AGs also protects epiphytes from drought stress during the dry season [8].

The ant-epiphyte association is initiated when AG ants collect seeds of AG epiphytes, carry them to their nests and incorporate them into the carton walls [1], [14], [15]. But most AG seeds lack typical adaptations for ant-dispersal, such as elaiosomes—the seed-borne food rewards that mediate most temperate and sub-tropical ant-seed mutualisms [16], [17], [18]. Rather, most Neotropical AG seeds occur in fleshy fruits typical of vertebrate-dispersed seeds. One species (*Peperomia macrostachya* A. Dietr. (Piperaceae)) lacks fleshy fruit, but bears spikes of exposed sticky arillate seeds. *P. macrostachya* seeds are also sometimes eaten by vertebrates, and may stick to passing animals as well [1], [19]. Adhering fruit pulp, arils, or elaiosomes found on the seeds of the various AG seed species may serve as food rewards for ants that disperse the seeds. Indeed, Davidson [1] noted apparently undamaged AG seeds in *Camponotus femoratus* (Fabricius) and *Crematogaster levior* (Longino) brood chambers, where larvae may have fed upon putative food rewards.

Several observations suggest, however, that nutritional rewards alone cannot explain ant response to AG seeds. The common AG ant *C. femoratus* demonstrated preferences among AG seed species, but these preferences did not reflect supposed nutritional value of seed-borne rewards [1]. The AG ants *C. femoratus* and *Pachycondyla goeldii* (Forel) also collected seeds of AG epiphytes when fruit pulp and elaiosomes had been completely removed or after seeds had passed through a vertebrate digestive system [1], [14]. On the other hand, AG seeds with putative food rewards intact were under-utilized by generalist non-AG ants [1], [20].

Even among typical ant-dispersed seeds that do bear nutritious elaiosomes, there is evidence that chemical cues in the elaiosome are sufficient to elicit seed-carrying behavior; elaiosomes from taxonomically diverse seeds have been found to contain 1,2-diolein, which can elicit seed-carrying when applied to dummy seeds [16], [17], [21], [22].

AG ants might, therefore, use non-nutritive chemical cues to find and recognize AG seeds. In an analysis of the volatile composition of seeds from 10 AG epiphyte species in seven families, nine of the species contained the compound methyl 2-hydroxy-6-methylbenzoate (6-MMS), and all 10 released blends of four other phenolic volatiles in various combinations [23]. Just as 1,2-diolein is shared by elaiosomes and mediates temperate ant-seed interactions, the compounds shared among AG seeds might be the ones responsible for seed-carrying behavior. However, *C. femoratus* rarely retrieved seed dummies treated with the five volatiles, and the role of these compounds remains ambiguous (Table S1) [24]. Further studies of *P. macrostachya* indicated that a blend of terpenoid and phenolic volatiles, including 6-MMS but not the other previously identified compounds, elicited olfactory attraction but not seed-carrying in *C. femoratus* [5].

One striking feature of the AG symbiosis is the taxonomic diversity of its participants, and here we ask whether seeds of different AG plants use the same or different chemistry to elicit seed-carrying in the AG ant *C. femoratus*. First we identify additional compounds shared among AG seeds and absent among non-AG congeners. Second, we use a behavior-guided procedure to identify relevant extracts and chromatographic fractions of AG seeds, and find that different chemical fractions of different AG seed species elicit seed-carrying.

## Methods

### Study area and organisms

Studies were conducted in November, 2004 and October through December of 2005 and 2006, at the Centro de Investigación y Capacitación Río Los Amigos in Madre de Dios, Peru (located at 12°34′07"S, 70°05′57"W) where AGs constructed by the ant *C. femoratus* are abundant. Aggregations of two to 30 nests occurred along trails at an average interval of one nest every 30 meters. *C. femoratus* occupied more than 95% of AGs in floodplain and terra firme habitats (*n* = 168 AGs censused); the remainder were constructed by *Azteca* species. In *C. femoratus* gardens, 98% of the nests and 100% of nest aggregations also housed *Cr. levior,* an ant species that lives with *C. femoratus* in poorly understood symbiosis termed parabiosis [25].

Nine epiphyte species regularly occur in AGs at the field site, where a single nest typically hosts one to four plant species. The three species with the most available seeds were compared using behavior-guided extraction and fractionation. These species were *Peperomia macrostachya*, which occupied 91% of all censused gardens at the site; *Anthurium gracile* Lindl. (Araceae), in 19% of gardens; and *Codonanthe uleana* Fritsch (Gesneriaceae) in 7%. All three species are rare outside of AGs in the Amazon: of 674 *P. macrostachya* plants observed by Davidson [1] at a nearby site, only five individuals grew independently of AGs. Similarly, six of 261 *A. gracile* individuals and no *C. uleana* individuals were found outside of AGs [1].

Additional AG and non-AG seeds were collected at the Estación Biológica Cocha Cashu, Madre de Dios, Peru (EBCC, 11°52′S, 71°22′W), in October, 2004.

### Survey of seed chemistry

To identify AG-specific seed chemistry, we collected seeds of nine AG seed species and eight non-AG congeners in 2004 and 2005 (Tables 1 and 2), taking seeds directly from mature fruits or seed spikes. Any adhering fruit pulp was removed with clean forceps, but arils were left intact. Some seeds were extracted with hexane or ethyl acetate in the field, and the extract returned to NCSU for laboratory analysis. Other seeds were stored in 1.5 ml 95% ethanol for transport to the lab. There, the ethanol supernatant was decanted and extracted with 1.8 ml hexane. Water (0.2 ml) was added to separate the ethanol and hexane, the mixture was centrifuged at 1000 rpm for 1 min, the hexane removed, and the water-ethanol phase extracted two more times with 1.8 ml hexane. The seeds that had been stored in ethanol were also soaked for 20 min in 1 ml hexane, and all hexane extracts of both the ethanol supernatant and the seeds were combined and evaporated under a gentle stream of N_2_ to a concentration suitable for analysis. Extracts were analyzed using gas chromatography-mass spectrometry (GC-MS). Conditions of instrumental analysis varied slightly (e.g. oven temperature program and carrier gas flow rate) but the results remained comparable over the three years of the study; a description of typical conditions follows. The GC was an Agilent 6890N, coupled to an Agilent 5975 mass selective detector, operating with Agilent Productivity ChemStation software. Manual injections of 1 µl were performed in splitless mode (1 min purge). Analyses were run on a nonpolar column (DB-5MS, 30 m×250 µm×0.25 µm protected by 2 m of deactivated guard column), and the oven temperature went from 40°C (2 min) to 300°C (20 min) at a rate of 10°C/min. The temperature of the injector port was 300°C, and the carrier gas was He with a flow of 1.2 ml/min. The MS transfer line was held at 280°C. Compounds common to all or most AG seed samples were compared to the Wiley 7^th^ Edition/NIST 05 mass spectral database, and identifications were confirmed by coinjection with authentic standards on both nonpolar and polar columns and by identity of unknown and standard mass spectra. The polar column was an Alltech 20294 WAX, 30 m×250 µm×0.25 µm. In this case, the oven program went from 40°C (2 min) to 260°C (30 min) at a rate of 10°C/min. The injector port and transfer line were held at 280°C, and He was the carrier gas with a flow of 1.2 ml/min.

**Table 1:** Occurrence of seven volatile compounds in AG epiphytes.

Family	Species	Origin^a^	Year	Solvent^b^	Compound (percent abundance in complete extract)^c^							
					1	2	3	4	5	6	7	total mass (ng)
Araceae	*Anthurium gracile*	CC	2004	EtOH, hex	0.4	7.6	3.5	37	3.4	0.9	8.4	41750
		CICRA	2004	hex	0.5	2.1		3.5	0.4		1.5	80
		CICRA	2005	EtOAc	6.2	7.5	1.4	35.9	1.8	0.9	17.08	840
	*Philodendron megalophyllum*	CC	2004	EtOH, hex	tr	6.7	2.5	36.9	0.6	0.2	10.8	2200
		CICRA	2004	EtOH, hex	tr	13.7	1.1	16.8	0.4			740
Bromeliaceae	*Aechmea longifolia*	CICRA	2004	EtOH, hex	1.4	5.8	7.7	7.2	0.7	0.3	16.5	37090
		CICRA	2004	hex	6.7	0.8	1.1	0.1	0.08	tr	0.3	360
	*Aechmea mertensii*	CICRA	2004	EtOH, hex	0.2	4.9		2.2	2.1	0.5	8.2	5090
	*Epiphyllum phyllanthus*	CICRA	2004	EtOH, hex	0.6	11.4	2.7	22.9	0.6	1.7	27.9	7760
Cactaceae		CICRA	2004	hex	tr	1.2		1.6	0.1		2.3	60
Gesneriaceae	*Codonanthe uleana*	CC	2004	EtOH, hex	tr	2.5	1.2	17.6	0.3	tr	1.6	3930
		CICRA	2004	EtOH, hex		5	3	23.8	0.9	1	0.8	3130
		CICRA	2004	EtOH, hex		2.5	1.7	25.6	0.5	tr	2.1	3010
		CICRA	2005	EtOAc		1.9	0.7	30.3	0.3	tr	1.5	480
Moraceae	*Ficus paraensis*	CC	2004	EtOH, hex		3.4	1.2	0.3	0.2	0.1		140
Piperaceae	*Peperomia macrostachya*	CC	2004	EtOH, hex	1.2	0.5	5.6	1.6	0.2	0.2		7960
		CICRA	2004	EtOH, hex	0.5	0.9	0.4	0.8	0.3	0.2		2580
		CICRA	2004	hex	0.3	0.9		0.9	0.1	0.1		820
		CICRA	2005	hex	1.1	3.2		2.9	0.4			675
		CICRA	2005	hex	1.8	3.2	0.6	8.5	2	0.8		440
Solanaceae	*Markea ulei*	CICRA	2004	EtOH, hex	6	9.1	21.8	5.7	1.5	0.3	0.9	1440
		CICRA	2004	EtOH, hex	3.8	10	20.1	4.7	4	1.5	4.7	1780

a Origin: CC, Estacion Biologica Cocha Cashu; CICRA, Centro de Investigacion y Capacitacion Rio Los Amigos.

b Solvent: EtOH, hex: seeds were stored in ethanol, then ethanol and seeds were extracted with hexane; hex: seeds were extracted in hexane for 1 hr (2004) or 30 min (2005); EtOAC: seeds were extracted in ethyl acetate for 30 min.

c Compounds: **1**, 6-MMS; **2**, β-springene; **3**, α-springene; **4**, geranyllinalool; **5**, unknown #1; **6**, unknown #2; **7**, geranylgeraniol.

**Table 2:** Absence of prevalent AG compounds in non-AG epiphytes.

Family	Species	Origin^a^	Year	Solvent^b^	Compound (percent abundance in complete extract)^c^							
					1	2	3	4	5	6	7	total mass (ng)
Araceae	*Anthurium bonplandii*	CICRA	2004	EtOH, hex								
	*Anthurium clavigerum*	CICRA	2004	EtOH, hex								
Bromeliaceae	*Aechema* sp. 1	CICRA	2004	EtOH, hex								
	*Aechmea* sp. 2	CICRA	2004	EtOH, hex								
Gesneriaceae	*Codonanthe* sp. d	CICRA	2004	EtOH, hex								
		CICRA	2004	EtOH, hex								
		CICRA	2004	hex								
Moraceae	*Ficus maxima*	CC	2004	EtOH, hex								
Piperaceae	*Peperomia* sp. 1	CICRA	2004	EtOH, hex								
	*Peperomia* sp. 2	CICRA	2004	EtOH, hex								

a Origin: CC, Estacion Biologica Cocha Cashu; CICRA, Centro de Investigacion y Capacitacion Rio Los Amigos.

b Solvent: EtOH, hex: seeds were stored in ethanol, then ethanol and seeds were extracted with hexane; hex: seeds were extracted in hexane for 1 hr (2004) or 30 min (2005); EtOAC: seeds were extracted in ethyl acetate for 30 min.

c Compounds: **1**, 6-MMS; **2**, β-springene; **3**, α-springene; **4**, geranyllinalool; **5**, unknown #1; **6**, unknown #2; **7**, geranylgeraniol.

d *Codonanthe* sp. was collected from *Azteca* sp. gardens in which it was the only epiphyte species present. Though this species was identified by Vega *et al.* (2006) as *C. uleana*, it was morphologically distinct and its seeds were not retrieved by *C. femoratus* when offered.

### Behavior-guided extraction and fractionation

Seed extracts of three AG plant species, *A. gracile*, *C. uleana*, and *P. macrostachya*, were subjected to seed-carrying assays in the field in 2004 and 2005. To obtain each extract, a group of 100 seeds of a single species were soaked in 3 ml of GC-grade *n*-hexane, ethyl acetate, or methanol for 30 min. Any fleshy or gelatinous fruit material was removed from seeds prior to extraction, but arils were left intact.

To perform the seed-carrying assay, extracts of AG seeds, (or chromatographic fractions thereof, see below) were applied to other seeds that ants typically ignore (*Piper laevigatum* Kunth). Using a 10 µl glass syringe, each test seed was treated with one AG seed-equivalent of extract. Extract-treated seeds were paired with control seeds that had been treated with an equal amount of solvent. Pairs of seeds were presented within 5 cm of foraging trails of *C. femoratus* ants. Each pair of seeds was observed for 20 min and scored as carried or not carried. Seeds that were handled but dropped were scored as not carried; when ants did walk away with seeds they appeared determined to carry the seeds to the nest and could sometimes be followed for meters still grasping a seed.

To test ant preference for hexane and methanol extracts of *C. uleana* and *P. macrostachya* in 2004, we presented six test seeds at a time: one treated with each extract and one with each solvent, and we noted the order in which seeds were carried over 20 minutes. All trials were repeated with *C. femoratus* from three different colonies. For each trial, we assigned each test seed a preference rank from zero to five, where zero was least preferred/carried last, and five was most preferred/carried first. To avoid missing values, seeds that were not carried were assigned the lowest rank in any given trial, or if *n* seeds were not carried, each was assigned the mean of the lowest *n* ranks. Ant preference was tested with an ANOVA on ranks [26] using the rank order of seed preference, in PROC GLM in the SAS System for Windows, version 9.1 [27].

Crude extracts that strongly elicited seed-carrying behavior in 2005 were duplicated and subjected to chromatographic fractionation. For *P. macrostachya*, extracts in hexane were highly preferred [28]. For *C. uleana* and *A. gracile*, extracts made with ethyl acetate or methanol were carried more often than those made with hexane [28], but methanol extracts were intractable to fractionate in the field. Therefore, the following fractionation procedure was applied to hexane extracts of *P. macrostachya* and ethyl acetate extracts of *C. uleana* and *A. gracile*. Fifty seed equivalents of extract were applied to a normal-phase chromatographic column packed with 200 mg silica gel, and eluted with 3 ml each of the following solvents: hexane, 5, 10, 30, and 70% ethyl acetate in hexane, ethyl acetate, and methanol. Crude extracts in hexane were concentrated and applied directly to the column; extracts in ethyl acetate were evaporated to dryness in a clean glass vial containing a small amount of silica gel, which was then returned to the column and eluted in the same manner. Pure solvents were subjected to the same procedures for use as controls in seed-carrying assays and laboratory analyses. Fifteen seed equivalents from each fraction were applied to 15 test seeds, which were used in the seed-carrying assay with at least three different ant colonies. Remaining crude extract and fractions were retained for laboratory analysis. Two *A. gracile* extracts, two *P. macrostachya* extracts, and one *C. uleana* extract were subjected to this procedure.

### Chemical analysis of fractions

Each crude extract and fraction was analyzed using GC-MS, as described above. Specific fractions that had elicited seed-carrying were analyzed further, using methods dictated by the polarity of the crude extract and the fraction itself. Corresponding blanks were analyzed to confirm that detected compounds were of seed origin.

To test for the presence of sugars in the methanol fractions of *A. gracile* and *C. uleana*, samples were aliquoted into microreaction vials in volumes corresponding to 1, 0.2 or 0.01 seed equivalent, evaporated to dryness, and resuspended in 10 µl MSTFA (*N*-methyl-*N*-(trimethylsilyl)trifluoroacetamide) and 10 µl pyridine. Reactions were warmed to 60°C for 30 min. Sugar standards (D-(-)-fructose, D-(+)-glucose), were derivatized in the same manner with 1 µg sugar per reaction. One or 0.5 µl of the reaction was injected in the GC-MS, equipped with the nonpolar column and with the oven programmed from 50°C (1 min) to 300°C (20 min) at 15°C/min. The inlet was held at 280°C in splitless mode. Other GC-MS parameters were as described above.

To detect amino acids in *A. gracile* and *C. uleana* fractions, samples were sent to the Molecular Structure Facility at the University of California, Davis, for analysis on a Li-citrate based Beckman 6300 amino acid analyzer. Samples (10 to 20 seed equivalents) were dried, resuspended in 200 µl AE-Cys dilution buffer, vortexed, spun down, and 50 µl loaded on the analyzer.

### Chemicals

6-MMS (methyl 2-hydroxy-6-methylbenzoate, 92%) was synthesized and purified as previously described [23]. Geranyllinalool [(6*E*,10*E*)-3,7,11,15-tetramethyl-1,6,10,14-hexadecatetraen-3-ol, 95%] was obtained from Fluka. β-springene [(6*E*,10*E*)-7,11,15-trimethyl-3-methylenehexadeca-1,6,10,14-tetraene, 88%] and α-springene [(3*E*,6*E*,10*E*)-3,7,11,15-tetramethylhexadeca-1,3,6,10,14-pentaene, 70%] were provided by S. Schulz. Geranylgeraniol [(2*E*,6*E*,10*E*)-3,7,11,15-tetramethyl-2,6,10,14-hexadecatetraen-1-ol, 85%], vanillin (4-hydroxy-3-methoxy-benzaldehyde, 99%), 2,4-dihydroxyacetophenone [1-(2,4-dihydroxyphenyl)ethanone 99%] and 4,hydroxy-acetophenone [1-(4-hydroxyphenyl)ethanone, 99%] were all obtained from Sigma-Aldrich.

## Results

### The AG seed signature

**Figure 1:**
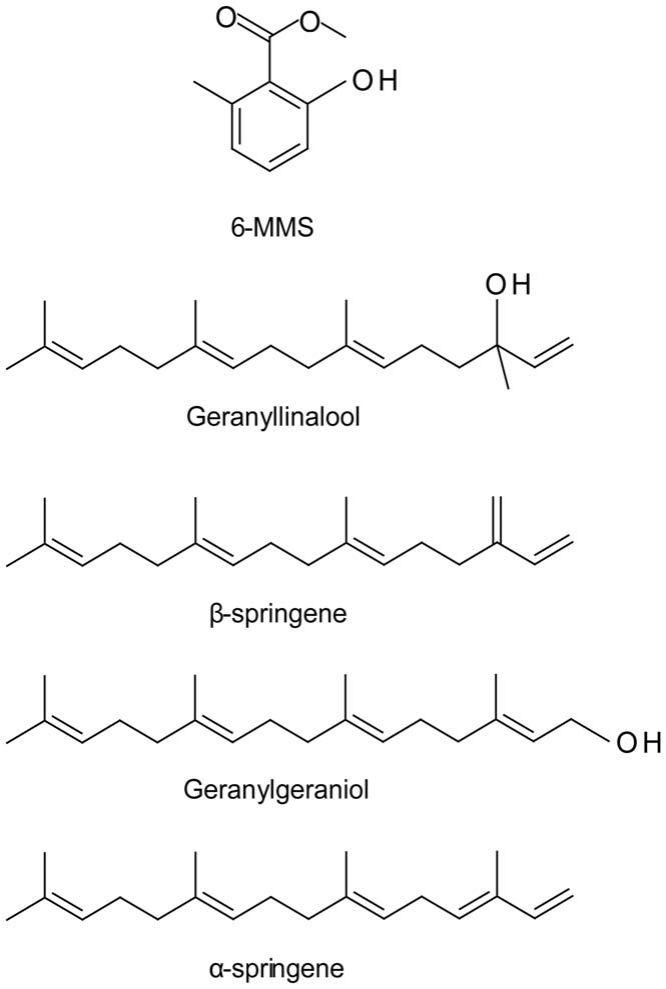
Structures of compounds frequently detected in AG seeds. These compounds were not detected in non-AG congeners (see Tables 1 and 2).

Seven compounds occurred frequently in AG seeds, but were absent in non-AG congeners (Fig. 1, Tables 1 and 2). Five of the seven compounds were identified as 6-MMS, α-springene, β-springene, geranyllinalool, and geranylgeraniol (for complete chemical names see "Chemicals" above). The remaining two compounds were not elucidated but were close structural relatives of one another, both characterized by electron impact mass spectra including a base peak at *m/z* 135, a putative molecular ion at *m/z* 272, and additional peaks at *m/z* 107, 93, 69 and 41. These compounds are probably allo-springenes derived from α- and β-springene [S. Schulz personal communication]. Both unknown compounds also occurred in synthetic α-springene and geranylgeraniol. All seven of the prevalent compounds occurred in widely varying amounts and ratios in the several AG species, ranging from barely detectable in some samples to the most abundant components of others (Table 1). Three of these compounds (β-springene, geranyllinalool, and one of the putative allo-springenes) were universally shared by all samples of all AG species analyzed; the other four compounds were widespread but not universal among AG seeds.

### Behavioral assays with crude extracts

**Figure 2:**
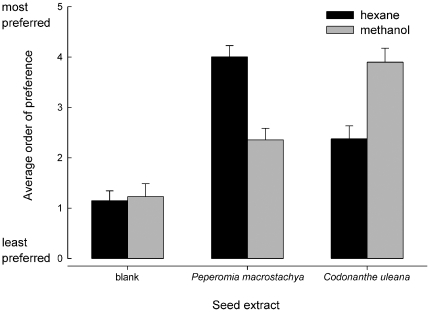
Ant response to hexane versus methanol extracts of AG seeds. AG ants (*C. femoratus*) preferred hexane extracts of *P. macrostachya* and methanol extracts of *C. uleana* in the seed-carrying assay. All extracts were preferred over solvent blanks. All six treatments were presented concurrently, and bars represent mean rank order in which seeds were carried during 24 30-min trials with three different ant colonies. Error bars are SEM. (Seeds that were carried last were assigned a rank of zero; a seed that was carried first was assigned a rank of five, and so on.)

Different solvents were optimal for extracting behaviorally relevant compounds from different AG seed species. When extracting *C. uleana*, it was noted that the seeds tended to clump together in hexane but not in ethyl acetate or methanol; *P. macrostachya* seeds, on the other hand, clumped in methanol but not hexane. In the seed-carrying assay, when ants could choose among hexane and methanol extracts of *C. uleana* and *P. macrostachya*, they preferred the methanol extract of *C. uleana* and the hexane extract of *P. macrostachya*, as evidenced by the significant ANOVA on ranks (Fig. 2; Table 3). The significant solvent by species interaction term confirms that extracting with methanol vs. hexane had opposite effects on ant preference for the two seed species considered. No variation in seed preference could be attributed to variation among the three ant colonies.

**Table 3:** Results of ANOVA on ranks, testing for effects of extract type (hexane or methanol extracts of *P. macrostachya*, *C. uleana*, or blanks) on the order in which ants retrieved test seeds.

Source	df	SS	*F*	*P*
Extract	5	184.4	25.8	<0.0001
Solvent x species interaction	1	60.2	42.1	<0.0001
Colony	2	0.0	0.0	1
Error	136	194.6		
Corrected total	143	379.0		

### Fractionation and analysis of fractions

**Figure 3:**
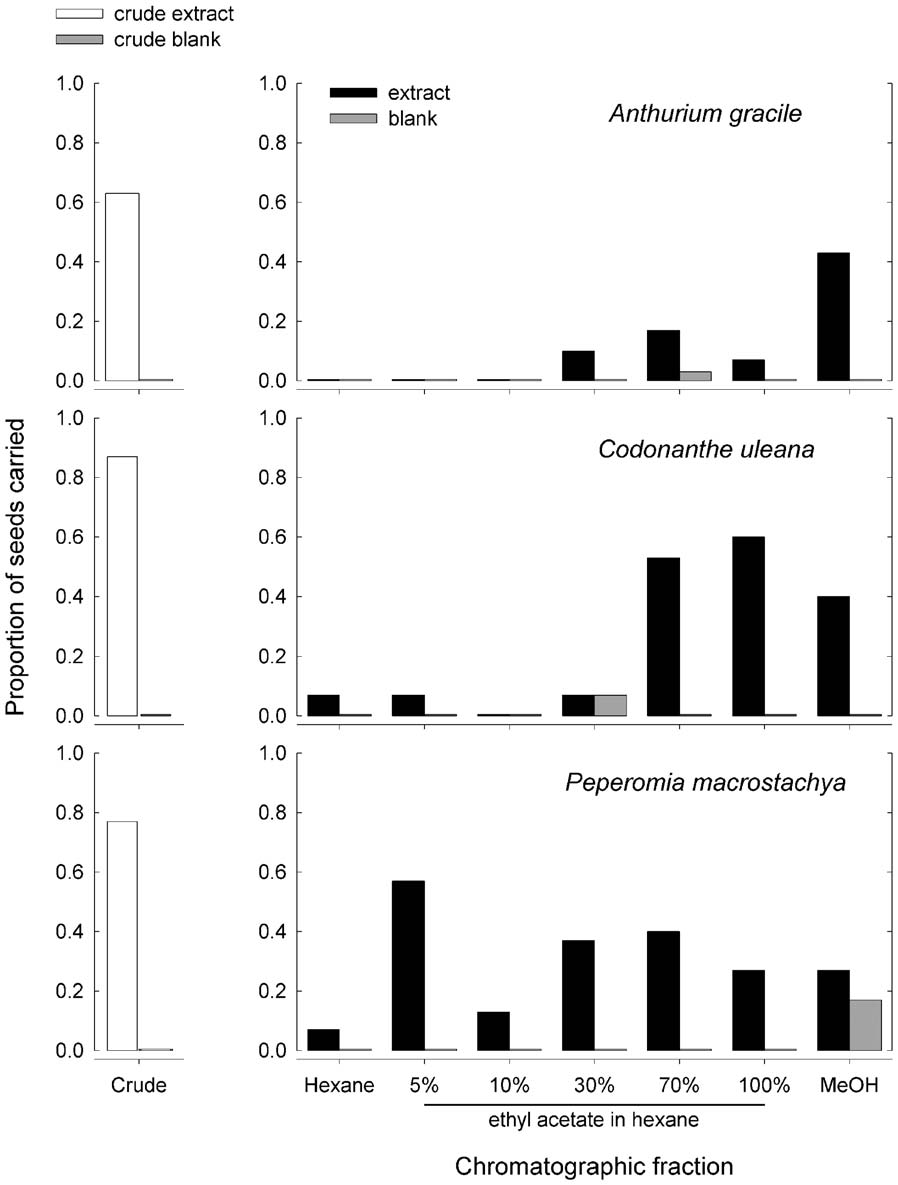
Ant response to chromatographic fractions of AG seed extracts. Ants preferred different chromatographic fractions of *A. gracile*, *C. uleana* and *P. macrostachya* extracts in the seed carrying assay. Seeds were extracted in hexane (*P. macrostachya*) or ethyl acetate (*A. gracile* and *C. uleana*) and the crude extract was tested in the seed carrying assay (left). Fractions of the crude extract were eluted successively with hexane, 5% to 70% ethyl acetate in hexane, ethyl acetate, and methanol (right). Proportions of seeds carried are out of 30 seeds for *A. gracile* and *P. macrostachya*, and out of 15 seeds for *C. uleana*. Each extract or fraction was tested with at least three different ant colonies.

Each crude extract that was subjected to chromatographic fractionation yielded at least one fraction that elicited seed-carrying in *C. femoratus* (Fig. 3). For hexane extracts of *P. macrostachya*, most fractions were somewhat active, but the 5% ethyl acetate fraction was nearly as effective as crude extract, particularly from one of the two extracts analyzed (Fig. 3 represents pooled data from all trials with both extracts). For *C. uleana*, the 70% ethyl acetate and 100% ethyl acetate fractions were most active; for *A. gracile*, only the methanol fraction elicited seed-carrying, but still resulted in less than 50% seed removal. Throughout the behavioral assays, ants sometimes handled seeds that they ultimately did not carry. (Blanks were very rarely handled.) We did not record the frequency with which different test seeds were handled, but we note that whenever ants manipulated seeds, regardless of whether they were ultimately carried, they did so using a combination of antennae, mouthparts, and front legs. All these appendages seemed to make fairly simultaneous contact with the seeds.

GC-MS analyses of the fractions confirmed that active fractions of different species differed in chemical composition. Shared compounds identified above as the “AG seed signature” did occur in the low-polarity fractions of all three species analyzed, but only in *P. macrostachya* did such fractions elicit seed-carrying behavior. Analysis of the 5% ethyl acetate fraction of *P. macrostachya* was described by Youngsteadt *et al.* [5].

The 70% ethyl acetate fraction of *C. uleana* contained vanillin, 4-hydroxyacetophenone, and 2,4-dihydroxyacetophenone. The identities of these compounds could not be confirmed with the 70% ethyl acetate fraction because this fraction was inadvertently destroyed after a preliminary analysis. The presence of these three compounds was, however, established in the original crude extract of *C. uleana* by coinjection and comparison of mass spectra with authentic standards. We could not detect anything in the 100% ethyl acetate fraction of the *C. uleana* extract, by GC-MS with or without derivatization with MSTFA (or with additional methods, see Discussion).

The 100% methanol fractions of *A. gracile* and *C. uleana* contained glucose and fructose (Table 4), confirmed by coinjection with authentic standards derivatized using the same methods. No sugars were detected in the methanol fraction of *P. macrostachya* using the same methods. The 100% methanol fraction of *A. gracile* also contained amino acids (Table 4).

**Table 4:** Sugar and amino acid content of methanol fractions of *A. gracile* and *C. uleana* extracts.

Sample	*A. gracile* 1	*A. gracile* 2	*C. uleana*
**Sugars (µg per seed)**			
Glucose	3.74	3.60±0.73 a	2.07
Fructose	2.29	5.83±0.37 a	2.89
**Amino acids (ng per seed)**			
aspartic acid	7.4		
threonine		7.4	
serine	1.7	18.1	
asparagine		13.4	
glutamic acid	6.8	7.4	
glycine		4.1	
alanine	2.5	12.6	
valine		8.9	
isoleucine		5.1	
leucine		13.6	
tyrosine	13.8	51.4	
phenylalanine		10.1	

a mean ± SD, 2 analyses.

## Discussion

Although AG plant species share a common interaction with mutualist ants, these results suggest that the different plant species elicit seed-carrying with different chemical cues. The seeds do share several volatile compounds, but we have not found evidence that these are important for ant behavior. Rather, for the different seed species examined, seed carrying is best obtained with extracts made from different solvents, or different chromatographic fractions of those extracts.

### Common compounds

We detected seven compounds (of which we identified five) in various combinations in all or most AG seeds. Six of the compounds, including the two unknowns, are structurally related terpenes or terpenoids; the seventh is phenolic. None of these was detected in seeds of non-AG congeners, though the terpene and terpenoid compounds have been identified in various other plant essential oils [29], [30], [31], [32]. All five identified compounds are known semiochemicals in other contexts, including as pheromones or components of defensive secretions in various hymenoptera and termites [33], [34], [35], [36].

6-MMS calls attention to itself because it has not been reported from plants other than the AG species; is also a semiochemical in various ant species; and occurs in the heads of male *C. femoratus* [23], [37], [38], [39], [40]. Indeed, Seidel *et al*. [23] suggested that the presence of 6-MMS in AG seeds might prompt *C. femoratus* workers to carry the seeds as if they were male brood.

Both geranyllinalool and 6-MMS elicited electrophysiological response from *Camponotus* antennae, and were part of an olfactorily attractive blend identified from the 5% ethyl acetate fraction of *P. macrostachya* [5]. In that blend, however, 6-MMS and geranyllinalool were mixed with three other phenolic compounds (3,5-dimethoxytoluene, methyl-*o*-anisate, and 3,5-dimethoxybenzoate) that did not occur in seeds of other AG species.

Despite the semiochemical potential of the five known compounds in the AG seed signature (6-MMS, α-springene, β-springene, geranylgeraniol and geranyllinalool) we were unable to find evidence that these compounds were important in the ant-seed behavioral interaction. A blend of these compounds in seed-like proportions was not olfactorily attractive to *C. femoratus* at either of two different relevant concentrations [28]. We did not test this blend alone in seed carrying assays; rather, these compounds were included with additional volatiles in an early attempt to create the most complete seed-like blends possible. Neither of two seed-like blends, based on 2004 analyses of crude extracts of *A. gracile* and *C. uleana*, elicited seed-carrying (Tables S1 and S2). Preliminary seed-carrying experiments with geranyllinalool and 6-MMS at a range of concentrations also suggested that these compounds alone did not elicit seed-carrying behavior (Tables S1 and S2). Given the variation in amounts and ratios of compounds among seed extracts and species (Table 1), it is unlikely that errors in concentrations or ratios in the synthetic blends would account for the lack of ant response. Alternatively, impurities in the synthetic compounds may have been problematic. Our synthetic geranyllinalool was certainly a racemic mixture, and our α-springene and β-springene standards were of moderate purity [28]. Finally, the compounds in the AG seed signature might still be important in the ant-seed interaction, at some stage other than seed-carrying (e.g. they might be part of an olfactorily attractive blend, or might influence how seeds are handled after ants return to the nest). These are issues to address in future studies; in the present study, we abandoned work with unpromising synthetic compounds in favor of behavior-guided fractionation of highly active seed extracts.

### Extracts and fractions

The results of the behavior-guided extraction and fractionation support the interpretation that compounds shared among AG seeds are not central to seed-carrying behavior in *C. femoratus*. The highly polar solvent methanol produced preferred extracts of *C. uleana*, whereas a nonpolar solvent (hexane) produced the preferred extracts of *P. macrostachya*. Similarly, the polar solvent ethyl acetate produced preferred extracts of both *C. uleana* and *A. gracile*, as compared to the nonpolar solvent hexane [28]. These observations were borne out in the fractionation results, where each species examined yielded a different pattern of behavioral activity among fractions. Although *P. macrostachya* was extracted with hexane and the other two species with ethyl acetate, all extracts did contain the shared compounds identified as the AG seed signature. The hexane fractions always included the springenes and putative allo-springenes (among other compounds), but were never preferred in seed-carrying assays. The remaining shared compounds eluted in low-polarity fractions, but only in *P. macrostachya* did such fractions elicit seed-carrying. In contrast, as suggested by ant preference for polar solvent extracts of *A. gracile* and *C. uleana*, more polar fractions of these extracts were also preferred in the seed-carrying assay.

Although the extraction and fractionation results indicate that different seed species use different classes of compounds to elicit seed-carrying behavior, the identities of the specific compounds responsible remain elusive. Monosaccharides (glucose and fructose) were present in the methanol fractions of *A. gracile* and *C. uleana*, and amino acids were also present at fairly low concentrations in *A. gracile*. Nevertheless, amounts on seeds were probably comparable to what ants might obtain in a drop of extrafloral nectar, especially since our ethyl acetate extracts would have removed only a small proportion of the sugars present on the seed surface. Sugars and amino acids are components of plant and hemipteran exudates that typically recruit ants, and sugar and amino acid composition can contribute to species specificity and species sorting in ant-plant interactions [41], [42]. We did not, however, find evidence that seed sugars have a role in the AG ant-seed interaction. Monosaccharides detected in seed extracts (glucose and fructose) as well as disaccharides (sucrose) were offered on test seeds alone, together, combined with the same amino acids detected on *A. gracile* seeds, or combined with the attractive 5-component blend described by Youngsteadt *et al*. [5]. These seeds were almost never carried (Table S3), and addition of glucose, fructose, or sucrose, together or separately, to dilute seed extracts did not increase ant preference for test seeds treated with those extracts (Fig. S1; Tables S4 and S5).

We made no attempt to behaviorally test the three phenolic volatiles tentatively identified from the 70% ethyl acetate fraction of *C. uleana*. However, both vanillin and 2,4-dihydroxyacteophenone had been previously detected in six and five species of AG seeds, respectively, and did not elicit consistent seed-carrying in previous studies [23], [24] or in preliminary tests performed in 2004 (Table S1). We could not detect anything in the 100% ethyl acetate fraction of *C. uleana* using the described methods, nor with HPLC-MS, nor with GC-MS after transmethylation to detect fatty acids.

Clearly, behaviorally relevant compounds are present in the seed extracts, and particularly in the active fractions, despite the fact that we were unable to pinpoint specific chemicals. This could be because active compounds are present in quantities below the detection limits of our instruments, because they were thermally unstable, highly polar, nonvolatile, or some combination of these characteristics. Additional methods for extracting, partitioning extracts, and analyzing fractions should eventually be brought to bear on this question.

Despite the lack of compound IDs, we conclude based on the extraction and fractionation results that different seed species use different classes of compounds to elicit seed-carrying behavior. It is reasonable that the taxonomically diverse AG species should have arrived at their interaction with ants through different biochemical pathways—but this leaves the role of the shared compounds unexplained. The lack of behavioral response to shared compounds does not support previously proposed hypotheses that AG ants carry AG seeds because the seeds resemble ant brood [19], [23]. If this were the case, one would expect all seeds to share a common brood signature. But the notion cannot be completely dismissed until more is known about how AG ants do, in fact, recognize brood.

The present results also contrast with other systems of seed dispersal by ants, where some 3,000 plant species in more than 80 families, mainly in the temperate and subtropical zones, have converged upon similar morphological and chemical adaptations for ant recruitment. Those that have been analyzed typically contain 1,2-diolein, a compound that elicits seed-carrying by mutualist ants, as well as a nutrient composition that differs markedly from that of the associated seed [16], [21], [22].

Our results are, on the other hand, reminiscent of those obtained for Southeast Asian AGs. There, solvent extracts of seeds were also sufficient to elicit seed-carrying in most AG ant species, but the specific compounds responsible were not identified. No common compounds were detected among AG seed extracts, suggesting that—as we also conclude for Neotropical AGs—the taxonomically diverse Southeast Asian AG plant species rely upon different compounds to elicit retrieval by ants [43]. Southeast Asian AG plants include both primarily bird-dispersed and wind-dispersed seeds, and Kaufmann [43] further suggested that those two classes of seeds probably use different chemical strategies, since the former were widely attractive to many ants, including non-AG species, while the latter were not.

Future studies of Neotropical AGs should revisit the role of the common compounds in the behavior of AG ants, after elucidating chirality of geranyllinalool and obtaining high-purity synthetic standards. Finally, the identity of seed-carrying cues remains to be determined. Though behavior-guided fractionation is a promising approach to address this question, active fractions may need to be further fractionated and analyzed by methods such as HPLC-MS to pinpoint nonvolatile or thermally unstable compounds that could have a role in the interaction.

## Supporting Information

Figure S1 **Ant response to (a) dilute** ***A. gracile*** **extract (0.1 seed-equivalent per test seed) alone or with the addition of glucose (G) and fructose (F), sucrose (S) or a combination of the three.** Addition of sugars did not enhance ant preference for test seeds. Because sugars were presented by weight rather than by their respective molarity, non -preference for sucrose could have resulted from fewer moles of sugar per seed, despite equal weight of sugar applied. Therefore, we performed an additional test in which moles of sugar per seed were held constant and mass of sucrose per seed was doubled (b). In each test, all treatments were presented concurrently, and bars represent mean rank order in which seeds were carried during fifteen 20-minute trials with three different ant colonies. Seeds that were carried last were assigned a rank of zero. Error bars are SEM.
(PDF)

Table S1 **Results of exploratory seed-carrying assays with volatile compounds, presented individually and in blends.**
(PDF)

Table S2 **Composition of blends mentioned in Table S1.**
(PDF)

Table S3 **Results of exploratory seed-carrying assays with sugars and with combinations of sugars, amino acids and volatile compounds.**
(PDF)

Table S4 **Results of ANOVA on ranks, testing for effects of treatment (dilute** ***A. gracile*** **extract alone or with the addition of glucose and fructose, sucrose or a combination of the three, matched for weight of sugar per seed) on the order in which ants retrieved test seeds.**
(PDF)

Table S5 **Results of ANOVA on ranks, testing for effects of treatment (dilute** ***A. gracile*** **extract alone or with the addition of glucose and fructose or sucrose, matched for moles of sugar per seed) on the order in which ants retrieved test seeds.**
(PDF)
