# Supplementary figures and images for: Divergent Chemical Cues Elicit Seed Collecting by Ants in an Obligate Multi-Species Mutualism in Lowland Amazonia
Source: PLoS One. 2010 Dec 30;5(12):e15822. doi: 10.1371/journal.pone.0015822 (PMC3012710; doi:10.1371/journal.pone.0015822)

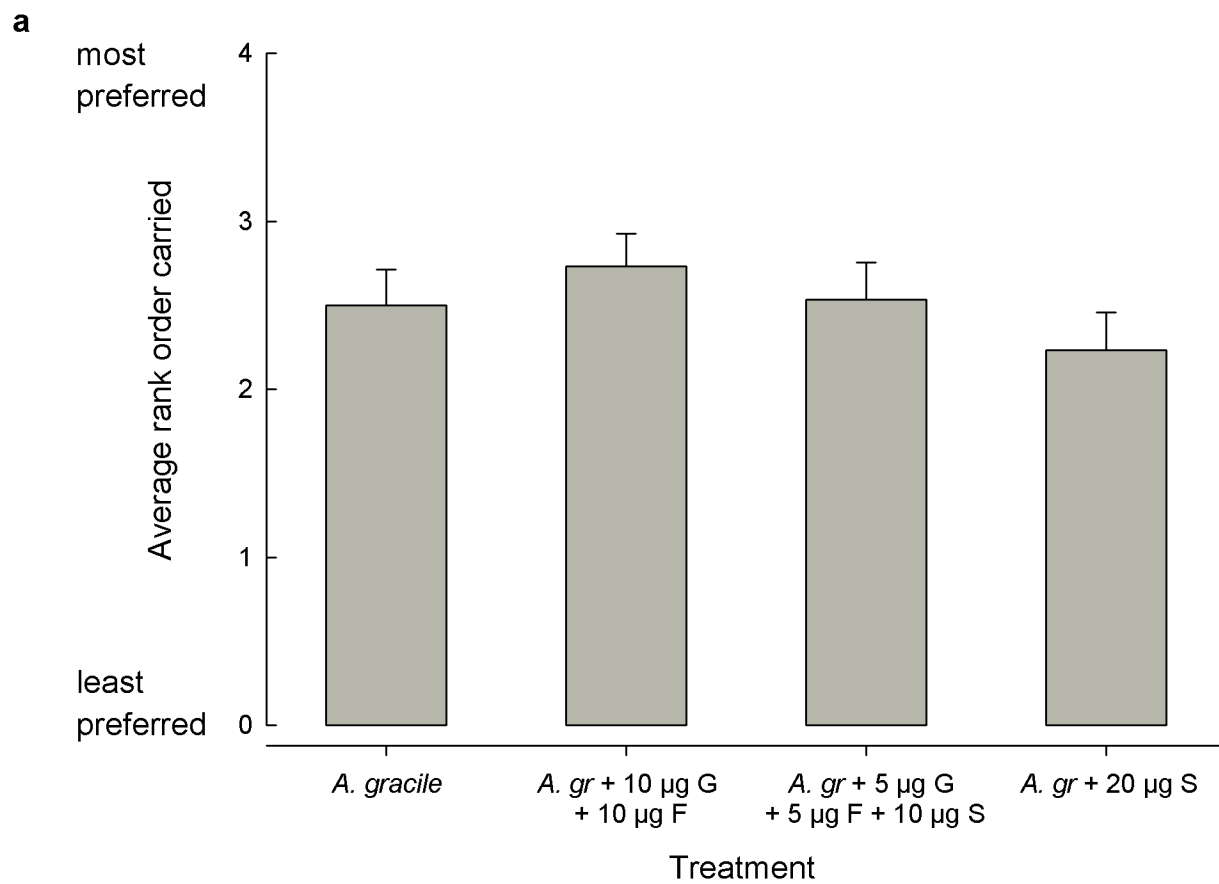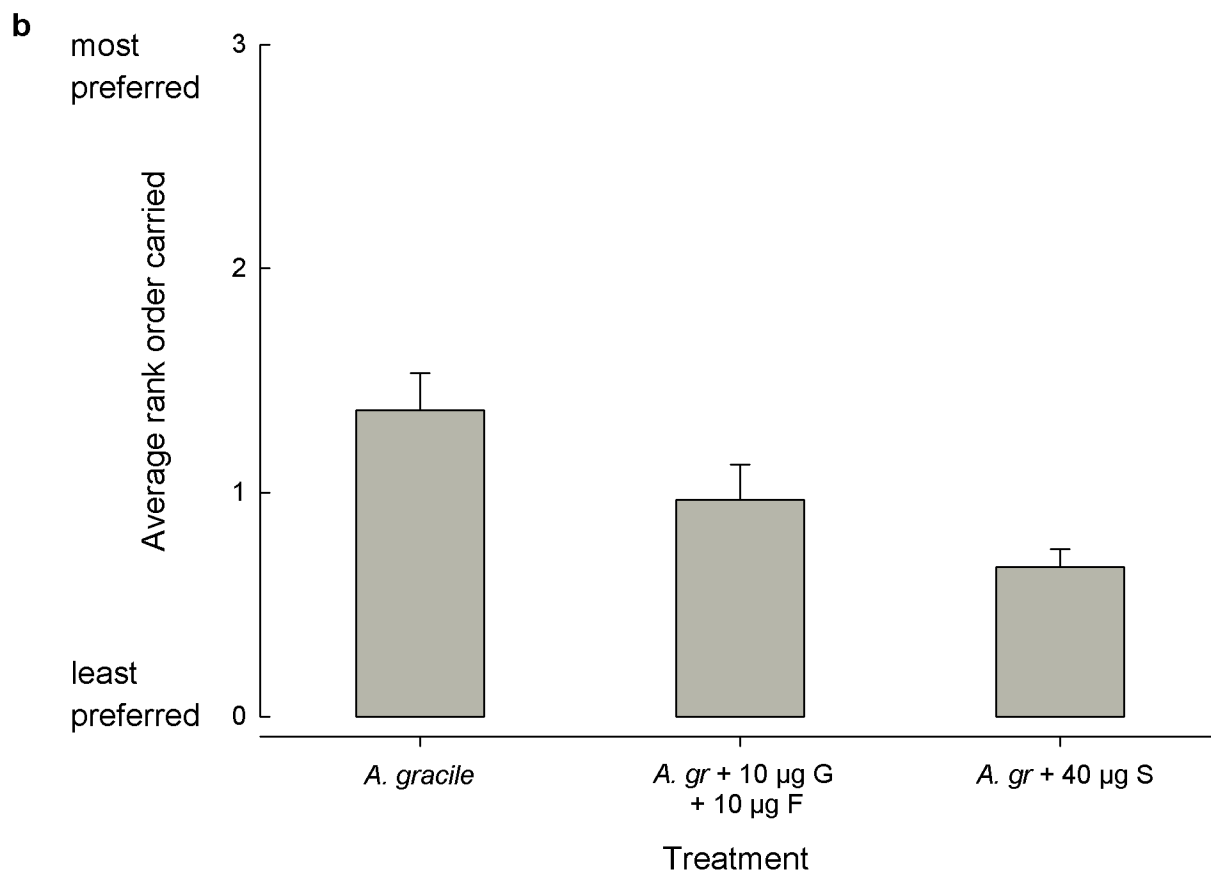

Supplement: Figure S1 — Ant response to (a) dilute A. gracile extract (0.1 seed-equivalent per test seed) alone or with the addition of glucose (G) and fructose (F), sucrose (S) or a combination of the three. Addition of sugars did not enhance ant preference for test seeds. Because sugars were presented by weight rather than by their respective molarity, non -preference for sucrose could have resulted from fewer moles of sugar per seed, despite equal weight of sugar applied. Therefore, we performed an additional test in which moles of sugar per seed were held constant and mass of sucrose per seed was doubled (b). In each test, all treatments were presented concurrently, and bars represent mean rank order in which seeds were carried during fifteen 20-minute trials with three different ant colonies. Seeds that were carried last were assigned a rank of zero. Error bars are SEM. (PDF) [file pone.0015822.s001.pdf]
