# Supplementary material for: Divergent Chemical Cues Elicit Seed Collecting by Ants in an Obligate Multi-Species Mutualism in Lowland Amazonia
Source: PLoS One. 2010 Dec 30;5(12):e15822. doi: 10.1371/journal.pone.0015822 (PMC3012710; doi:10.1371/journal.pone.0015822)
Supplement: Table S1 — Results of exploratory seed-carrying assays with volatile compounds, presented individually and in blends. (PDF) [file pone.0015822.s002.pdf]

Table S1. Results of exploratory seed-carrying assays with volatile compounds, presented individually and in blends.

Compound or blend	Amount (µg per seed) [a]	Seeds presented **total** (# per colony)	Seeds carried **total** (# per colony)	Blanks presented **total** (# per colony)	Blanks carried **total** (# per colony)	Location [b]	Date
6-MMS	10	**24** (3,6,3,6,3,3)	**1** (1,0,0,0,0,0)	**24** (3,6,3,6,3,3)	**0**	CC	October, 2004
	50	**26** (5,12,3,3,3)	**2** (0,1,0,1,0)	**26** (5,12,3,3,3)	**2** (1,1,0,0,0)	CC	October, 2004
	100	**18** (3,3,6,3,3)	**1** (0,0,0,1,0)	**18** (3,3,6,3,3)	**3** (0,0,3,0,0)	CC	October, 2004
Benzothiazole	10	**22** (6,8,3,5)	**2** (2,0,0,0)	**22** (6,8,3,5)	**1** (1,0,0,0)	CC	October, 2004
	50	**19** (3,8,3,5)	**0**	**19** (3,8,3,5)	**2** (0,2,0,0)	CC	October, 2004
	100	**22** (6,8,3,5)	**0**	**22** (6,8,3,5)	**3** (1,2,0,0)	CC	October, 2004
Vanillin	10	**23** (11,6,3,3)	**7** (2,1,2,2)	**23** (11,6,3,3)	**0**	CC	October, 2004
	50	**36** (13,9,6,3,3,2)	**12** (5,3,3,0,1,0)	**36** (13,9,6,3,3,2)	**1** (1,0,0,0,0,0)	CC	October, 2004
	100	**21** (3,3,6,3,3,3)	**2** (0,1,0,0,1,0)	**21** (3,3,6,3,3,3)	**1** (0,0,0,1,0,0)	CC	October, 2004
1-(2-Hydroxy-6-	10	**9** (3,3,3)	**0 0**	**9** (3,3,3)	**1** (0,0,1)	CC	October, 2004
methylphenyl)	50	**15** (6,6,3)	**0 0**	**15** (6,6,3)	**0**	CC	October, 2004
ethanone	100	**15** (6,6,3)	**0 0**	**15** (6,6,3)	**0**	CC	October, 2004
2,4-Dihydroxy-	10	**20** (3,6,3,8)	**3** (2,0,0,1)	**20** (3,6,3,8)	**4** (0,2,0,2)	CC	October, 2004
acetophenone	50	**20** (3,6,3,8)	**1** (1,0,0,0)	**20** (3,6,3,8)	**0**	CC	October, 2004
	100	**20** (3,6,3,8)	**2** (0,1,0,1)	**20** (3,6,3,8)	**1** (1,0,0,0)	CC	October, 2004
Geranyl linalool	10	**12** (5,5,2)	**0**	**12** (5,5,2)	**0**	CICRA	October, 2005
	100	**10** (5,5)	**1** (1,0)	**10** (5,5)	**0**	CICRA	October, 2005
**Blends** [c]							
A. gracile -like	80	**15** (5,5,5)	**3** (2,1,0)	**15** (5,5,5)	**1** (1,0,0)	CICRA	October, 2005
blend	240	**5** (5)	**1** (1)	**5** (5)	**0**	CICRA	October, 2005
C. uleana -like	10	**15** (5,5,5)	**1** (1,0,0)	**15** (5,5,5)	**0**	CICRA	October, 2005
blend							
EAD blend	0.16	**20** (10,5,5)	**0**	**20** (10,5,5)	**0**	CICRA	October, 2006
	1.6	**35** (15,10,5,5)	**3** (3,0,0,0)	**35** (15,10,5,5)	**0**	CICRA	October, 2006
	16	**15** (5,5,5)	**1** (1,0,0)	**15** (5,5,5)	**0**	CICRA	October, 2006

All treatments were applied to *P. laevigatum* seeds, were paired with solvent-treated controls and were observed for 20 min., as described for other seed-carrying assays in the main text. The first five compounds are those that Seidel et al. (1990) identified as prevalent among AG seeds.

[a] One seed-equivalent was generally the starting point, with additional concentrations calculated as multiple seed-equivalents. Amount of compounds in one seed-equivalent was based on the most current analyses of seed extracts available at the time the assays were performed. In 2004, concentrations were based on Seidel et al. (1990) and Davidson et al. (1990).

[b] Location: CC = Estacion Biologica Cocha Cashu; CICRA = Centro de Investigación y Capacitación Rio Los Amigos

[c] Blend compositions are detailed in Table S2.
