# Supplementary material for: Divergent Chemical Cues Elicit Seed Collecting by Ants in an Obligate Multi-Species Mutualism in Lowland Amazonia
Source: PLoS One. 2010 Dec 30;5(12):e15822. doi: 10.1371/journal.pone.0015822 (PMC3012710; doi:10.1371/journal.pone.0015822)
Supplement: Table S2 — Composition of blends mentioned in Table S1. (PDF) [file pone.0015822.s003.pdf]

Table S2. Composition of blends mentioned in Table S1.

Compound	μg per seed-equivalent		
	A. gracile -like blend	*C. uleana* -like blend	EAD blend
Geranyllinalool	48.00	6.00	0.1
Geranylgeraniol	12.00	0.39	
Palmitic acid, ethyl ester	7.00		
Beta springene	4.00	0.30	
Linoleic acid, ethyl ester	4.00	0.05	
Alpha springene	2.00	0.20	
Oleic acid, ethyl ester	2.00	0.03	
6-MMS	0.40		0.02
Cembrene	0.40	0.01	
Heneicosane	0.08	0.02	
Hexadecanal	0.06	0.02	
Heptacosane	0.06	0.01	
Tridecane	0.04		
1-Tetradecene	0.04		
Beta caryophyllene	0.04		
Squalene	0.04	3.00	
Tricosane	0.04	0.01	
Pentadecane	0.02		
3,5-Dimethoxytoluene			0.02
Methyl anisate			0.01
3,5-Dimethoxybenzoate			0.01

A. gracile - and *C. uleana* -like blends were based on 2004 GC-MS analyses of hexane extracts of seeds and ethanol in which seeds had been stored, as described in the main text. The EAD blend was based on GC-MS and GC-EAD analyses of a fraction of *P. macrostachya* extract, as described by Youngsteadt et al. (2008). Blends were made from standard solutions in batches of 20 to 100 seed-equivalents each.
