# Supplementary material for: Divergent Chemical Cues Elicit Seed Collecting by Ants in an Obligate Multi-Species Mutualism in Lowland Amazonia
Source: PLoS One. 2010 Dec 30;5(12):e15822. doi: 10.1371/journal.pone.0015822 (PMC3012710; doi:10.1371/journal.pone.0015822)
Supplement: Table S3 — Results of exploratory seed-carrying assays with sugars and with combinations of sugars, amino acids and volatile compounds. (PDF) [file pone.0015822.s004.pdf]

Table S3. Results of exploratory seed-carrying assays with sugars and with combinations of sugars, amino acids and volatile compounds.

Amount per seed						Seeds presented	Seeds carried	Blanks presented	Blanks carried
Glucose (μg)	Fructose (μg)	Sucrose (μg)	Amino acid mix* (ng)	6-MMS (μg)	Geranyllinalool (μg)	**Total** (# per colony)	**Total** (# per colony)	**Total** (# per colony)	**Total** (# per colony)
5	5					**15** (5,5,5)	**0**	**15** (5,5,5)	**0**
2.5	2.5	5				**15** (5,5,5)	**0**	**15** (5,5,5)	**0**
20	20		200			**10** (5,5)	**2** (1,1)	**10** (5,5)	**0**
10	10	20	200			**10** (5,5)	**0**	**10** (5,5)	**0**
50	50					**13** (5,5,3)	**0**	**13** (5,5,3)	**0**
50	50		200			**13** (5,5,3)	**0**	**13** (5,5,3)	**0**
50	50		200	0.2	1	**13** (5,5,3)	**0**	**13** (5,5,3)	**0**
				0.2	1	**13** (5,5,3)	**0**	**13** (5,5,3)	**0**

Treatments were applied to *P. laevigatum* seeds and were paired with solvent-treated controls. Seeds were observed for 20 min., as described in for other seed-carrying assays the main text. All assays were performed at CICRA in October, 2006.

*The amino acid mix contained equal parts of alanine, glutamic acid, serine, threonine, asparagine, glycine, valine, isoleucine, phenylalanine, and tyrosine.
