# Supplementary material for: Divergent Chemical Cues Elicit Seed Collecting by Ants in an Obligate Multi-Species Mutualism in Lowland Amazonia
Source: PLoS One. 2010 Dec 30;5(12):e15822. doi: 10.1371/journal.pone.0015822 (PMC3012710; doi:10.1371/journal.pone.0015822)
Supplement: Table S4 — Results of ANOVA on ranks, testing for effects of treatment (dilute A. gracile extract alone or with the addition of glucose and fructose, sucrose or a combination of the three, matched for weight of sugar per seed) on the order in which ants retrieved test seeds. (PDF) [file pone.0015822.s005.pdf]

Table S4. Results of ANOVA on ranks, testing for effects of treatment (dilute *A. gracile* extract alone or with the addition of glucose and fructose, sucrose or a combination of the three, matched for mass of sugar per seed) on the order in which ants retrieved test seeds.

Source	df	SS	*F*	*P*
Treatment	3	1.9	1.1	0.35
Colony	2	0.0	0.0	1.00
Treatment x Colony Interaction	6	11.3	3.4	<0.01
Error	48	26.8		
Total	59	40.0		
