# Supplementary material for: Divergent Chemical Cues Elicit Seed Collecting by Ants in an Obligate Multi-Species Mutualism in Lowland Amazonia
Source: PLoS One. 2010 Dec 30;5(12):e15822. doi: 10.1371/journal.pone.0015822 (PMC3012710; doi:10.1371/journal.pone.0015822)
Supplement: Table S5 — Results of ANOVA on ranks, testing for effects of treatment (dilute A. gracile extract alone or with the addition of glucose and fructose or sucrose, matched for moles of sugar per seed) on the order in which ants retrieved test seeds. (PDF) [file pone.0015822.s006.pdf]

Table S5. Results of ANOVA on ranks, testing for effects of treatment (dilute *A. gracile* extract alone or with the addition of glucose and fructose or sucrose, matched for moles of sugar per seed) on the order in which ants retrieved test seeds.

Source	df	SS	*F*	*P*
Treatment	2	0.5	1.1	0.35
Colony	2	0.0	0.1	0.92
Treatment x Colony Interaction	4	3.4	3.4	<0.05
Error	36	9.0		
Total	44	13.0		
